# Monthly Variation in the Macromolecular Composition of Phytoplankton Communities at Jang Bogo Station, Terra Nova Bay, Ross Sea

**DOI:** 10.3389/fmicb.2021.618999

**Published:** 2021-02-11

**Authors:** Kwanwoo Kim, Jisoo Park, Naeun Jo, Sanghoon Park, Hyeju Yoo, Jaehong Kim, Sang Heon Lee

**Affiliations:** ^1^Department of Oceanography, Pusan National University, Busan, South Korea; ^2^Division of Ocean Sciences, Korea Polar Research Institute, Incheon, South Korea

**Keywords:** Ross Sea (Antarctica), phytoplankton biomass, macromolecular composition, food material, polar night

## Abstract

Organic carbon fixed by photosynthesis of phytoplankton during the polar growing period could be important for their survival and consumers during the long polar night. Differences in biochemical traits of phytoplankton between ice-free and polar night periods were investigated in biweekly water samples obtained at the Korean “Jang Bogo Station” located in Terra Nova Bay, Antarctica. The average concentration of total Chl-*a* from phytoplankton dominated by micro-sized species from the entire sampling period was 0.32 μg L^–1^ (SD = ± 0.88 μg L^–1^), with the highest concentration of 4.29 μg L^–1^ in February and the lowest concentration of 0.01 μg L^–1^ during the ice-covered polar night (April–October) in 2015. The highest protein concentration coincided with the peak Chl-*a* concentration in February and decreased rapidly relative to the carbohydrate and lipid concentrations in the early part of polar night. Among the different biochemical components, carbohydrates were the predominant constituent, accounting for 69% (SD = ± 14%) of the total particulate organic matter (POM) during the entire study period. The carbohydrate contributions to the total POM markedly increased from 39 ± 8% during the ice-free period to 73 ± 9% during the polar night period. In comparison, while we found a significant negative correlation (*r*^2^ = 0.92, *p* < 0.01) between protein contributions and carbohydrate contributions, lipid contributions did not show any particular trend with relatively small temporal variations during the entire observation period. The substantial decrease in the average weight ratio of proteins to carbohydrates from the ice-free period (mean ± SD = 1.0 ± 0.3) to the ice-covered period (mean ± SD = 0.1 ± 0.1) indicates a preferential loss of nitrogen-based proteins compared to carbohydrates during the polar night period. Overall, the average food material (FM) concentration and calorific contents of FM in this study were within the range reported previously from the Southern Ocean. The results from this study may serve as important background data for long-term monitoring of the regional and interannual variations in the physiological state and biochemical compositions of phytoplankton resulting from future climate change in Antarctica.

## Introduction

In high latitude polar waters, the light availability of phytoplankton is limited to a short ice-free period during summer (Arrigo and Van Dijken, 2004; Borrione and Schlitzer, 2013). During this period, phytoplankton can synthesize particulate organic matter (POM) through photosynthesis and provide an important food source supporting almost the entire marine ecosystem from the ice-free period to the end of the ice-covered polar night (Falkowski, 1994; Fabiano et al., 1996). Ongoing climate change has caused a remarkable reduction in the Arctic sea ice extent and a small increase in sea ice in the Southern Ocean for several decades (Arrigo et al., 2008; Markus et al., 2009; Liu and Curry, 2010; Comiso, 2012; Hobbs et al., 2016). In addition to the changes in sea ice coverage, the timing and duration of sea ice cover are also changing (Hobbs et al., 2016; Eayrs et al., 2019). In the Southern Ocean, the annual ice-free period has shortened by 2.6 months in the Western Ross Sea but increased by 3.3 months in the Bellingshausen Sea (Parkinson, 1994, 2002; Stammerjohn et al., 2008, 2012; Hobbs et al., 2016; Eayrs et al., 2019). These variations in the ice-free period can affect the duration of the growing season of phytoplankton as well as the nutritional conditions of upper trophic grazers (Quetin et al., 2007; Arrigo et al., 2008; Ross et al., 2008; Markus et al., 2009; Quetin and Ross, 2009; Massom et al., 2013).

Previous studies on the biochemical composition of phytoplankton in the Southern Ocean have been mainly conducted during the Austral summer (Fabiano et al., 1995; Fabiano and Pusceddu, 1998; Kim et al., 2016, 2018; Song et al., 2016), which provides essential information on the physiological state of phytoplankton during the ice-free period. However, these studies do not cover how the biochemical composition changes after the ocean is covered with sea ice again. As mentioned earlier, the ice-free and ice-covered periods and timing of each period vary locally in the Southern Ocean under the potential influence of climate change (Stammerjohn et al., 2008, 2012; Hobbs et al., 2016; Eayrs et al., 2019). These variations could affect the biomass and physiological state of phytoplankton.

The Jang Bogo Station (JBS) is the second overwintering research station of South Korea, located in Terra Nova Bay (TNB) in the Ross Sea (74° 37.4′ S, 164° 12.0′ E). The presence of thick seasonal sea ice and the absence of light during the polar night period limit access of researchers to the coastal region of the Southern Ocean, but the JBS provides the research opportunity during the polar night period. Previous studies have focused on the ice-free period in the TNB (Gambi et al., 2000; Majewska et al., 2013; Illuminati et al., 2017). Our study period covers almost an entire year from the ice-free period in February to the ice-covered polar night period in October 2015. Our objectives in this study were to investigate the biochemical characteristics of phytoplankton and to evaluate the calorific content of phytoplankton as the primary food source during the ice-free and ice-covered periods at the JBS in the TNB, Ross Sea. This study is important as a basis for future studies to understand the impact of current climate change on phytoplankton in the Antarctic Ocean.

## Materials and Methods

### Study Area and Water Sampling

**FIGURE 1:**
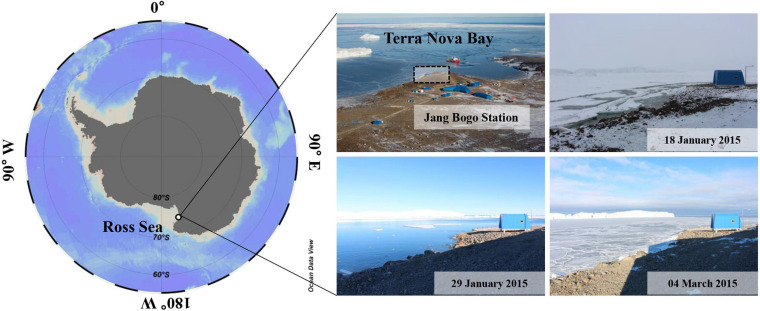
Study area and photos for the sampling station at JBS, 2015. A dashed box is a sampling location in our study.

Sample collection was performed at one fixed station (74°37′39.59″S, 164°14′25.75″E) near the Jang Bogo Station (JBS) located in Terra Nova Bay (TNB), Antarctica, from 2 February to 20 October 2015 (Figure 1). The polar night period was from 7 May to 7 August, and we divided the whole study period between the ice-free period (February) and the ice-covered period (April–October), including the polar night.

Surface water samples were collected with a 5 L Niskin water sampler when the coastal area was opened in February. During the ice-covered period, sampling was performed from a tank where the under-ice seawater (4–7 m below the sea ice) was continuously pumped. The tank had an inflow of surface seawater, and fresh surface seawater was always supplied in it by an overflowing system. Water samples were placed in dark containers and moved to the laboratory for filtration and further analysis.

### Chlorophyll *a* Analysis

Seawater samples for total chlorophyll *a* (Chl-*a*) concentration were filtered onto 25 mm GF/F filters (Whatman, nominal 0.7 μm pore size). For size-fractionated Chl-*a*, the samples were passed sequentially through 20 μm and 2 μm membrane filters and then 47 μm GF/F filters (Whatman, nominal 0.7 μm) with gentle pressure. Chl-*a* was extracted in 90% acetone for 24 h at 4°C following Parsons et al. (1984) and quantified using a Trilogy fluorometer (Turner Designs, United States), which had been calibrated with commercially purified Chl-*a* preparations.

### Analysis of Particulate Organic Carbon, Nitrogen, and δ^13^C

Water samples for particulate organic carbon (POC), nitrogen (PON), and δ^13^C of the total microbial community (>0.7 μm) were collected and filtered onto pre-combusted GF/F filters (25 mm) from April to October 2015. In the case of pico-sized POC and PON (0.7–2 μm), samples were filtered through a 2 μm pore-sized membrane filter, and then the filtrates were filtered onto pre-combusted GF/F filters (25 mm). The filters were immediately frozen and preserved at −80°C until analysis. We obtained the POC, PON, and isotopic values of filtered samples from a Finnigan Delta^*plus*^ XL mass spectrometer at the Stable Isotope Laboratory of the University of Alaska Fairbanks. The final concentrations of POC and PON were derived by dividing the volume of the filtered water sample.

### Analysis of Macromolecular Compositions of Phytoplankton

In order to determine macromolecular compositions of total POM, water samples were filtered onto 47 mm GF/F filters (nominal pore size = 0.7 μm) and stored at −80°C until analysis. In the case of pico-sized POM, water samples were passed sequentially through 2 μm membrane filters and 47 mm GF/F filters (nominal pore size = 0.7 μm) before being stored at −80°C. Extractions and quantifications of macromolecular components were executed in the laboratory located at Pusan National University of South Korea following Bhavya et al. (2019). In brief, for the quantitative analysis of total carbohydrates, we used the phenol-sulfuric acid method described by Dubois et al. (1956) and used a glucose solution (1 mg mL^–1^, Sigma) as a standard. To measure other macromolecular components, we performed an experiment according to a method described by Lowry et al. (1951) for total proteins and Bligh and Dyer (1959) and Marsh and Weinstein (1966) for total lipids. We used a protein standard (2 mg mL^–1^, Sigma) and a tripalmitin solution (Sigma) to calculate the final concentrations of proteins and lipids, respectively. Each extracted biochemical component was quantified with a HITACHI UH5300 spectrophotometer. The carbon contents of each biochemical component were calculated using 0.40, 0.49, and 0.75 g C g^–1^ conversion factors (carbohydrates, proteins, and lipids, respectively) (Fichez, 1991a, b; Danovaro et al., 2000). The biopolymeric carbon (BPC) was defined as the sum of the carbon concentration of each parameter (Fichez, 1991a, b; Danovaro et al., 2000).

### Calculation of Food Material (FM) Concentration, Calorific Value of FM, and Calorific Content of FM

Food materials (FM) were defined as the sum of carbohydrate, protein, and lipid concentrations (Danovaro et al., 2000). We calculated the calorific value (Kcal g FM^–1^) and calorific content of FM (Kcal m^–3^) (Fabiano et al., 1993, 1996) according to the Winberg (1971) equation (Kcal g^–1^ = 0.041^∗^CHO% + 0.055^∗^PRT% + 0.095^∗^LIP%).

### Statistical Analysis

Statistical analyses were carried out with the software SPSS (version 22) for *t*-tests and Pearson’s correlation coefficient. We set the level of significance at *p* < 0.05.

## Results

### Particulate Organic Matter at JBS in 2015

**FIGURE 2:**
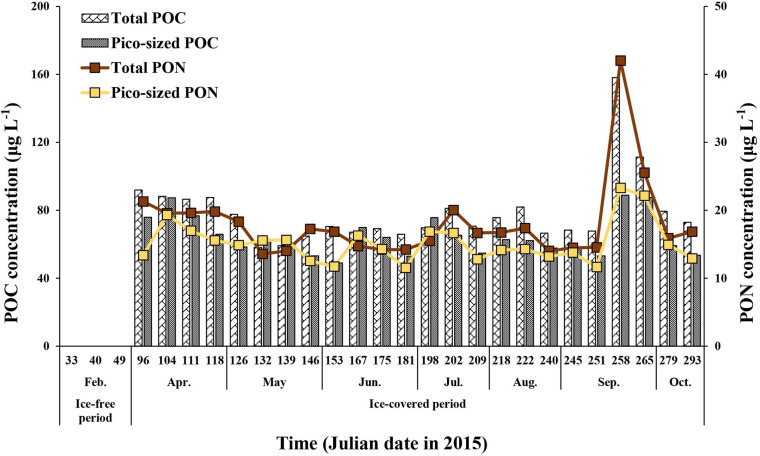
The total (>0.7 μm) and pico-sized (0.7–2 μm) POC and PON concentrations at JBS during the ice-covered period, 2015.

**FIGURE 3:**
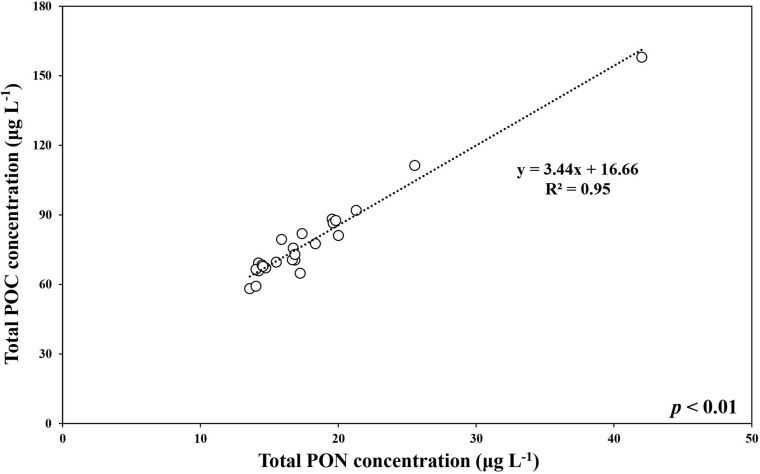
The relationship between total POC and total PON concentrations at JBS during the ice-covered period, 2015.

During the ice-covered period, the total POC concentration (>0.7 μm) ranged from 58.2 to 158.1 μg L^–1^ (mean ± SD = 78.8 ± 20.6 μg L^–1^), whereas the pico-sized POC concentration ranged from 49.0 to 88.8 μg L^–1^ (mean ± SD = 64.8 ± 11.6 μg L^–1^) (Figure 2 and Supplementary Tables 1, 2). The ranges for the total and pico-sized PON were between 13.6 and 42.0 μg L^–1^ (mean ± SD = 18.1 ± 5.9 μg L^–1^) and 11.5–23.3 μg L^–1^ (mean ± SD = 15.2 ± 3.0 μg L^–1^), respectively (Figure 2 and Supplementary Tables 1, 2). A strong linear relationship was found between total POC and PON concentrations in our study (POC = 3.44^∗^PON + 16.66, *r*^2^ = 0.95, *p* < 0.01) (Figure 3). Based on the POC and PON concentrations, the average C/N ratio during the entire study period was 5.15 with a small temporal variation (SD = ± 0.36) (Supplementary Table 1). The δ^13^C value of POC ranged from −30.83 to −27.17 ‰, with an average of −29.31% (SD = ± 1.11%) over the sampling period.

**FIGURE 4:**
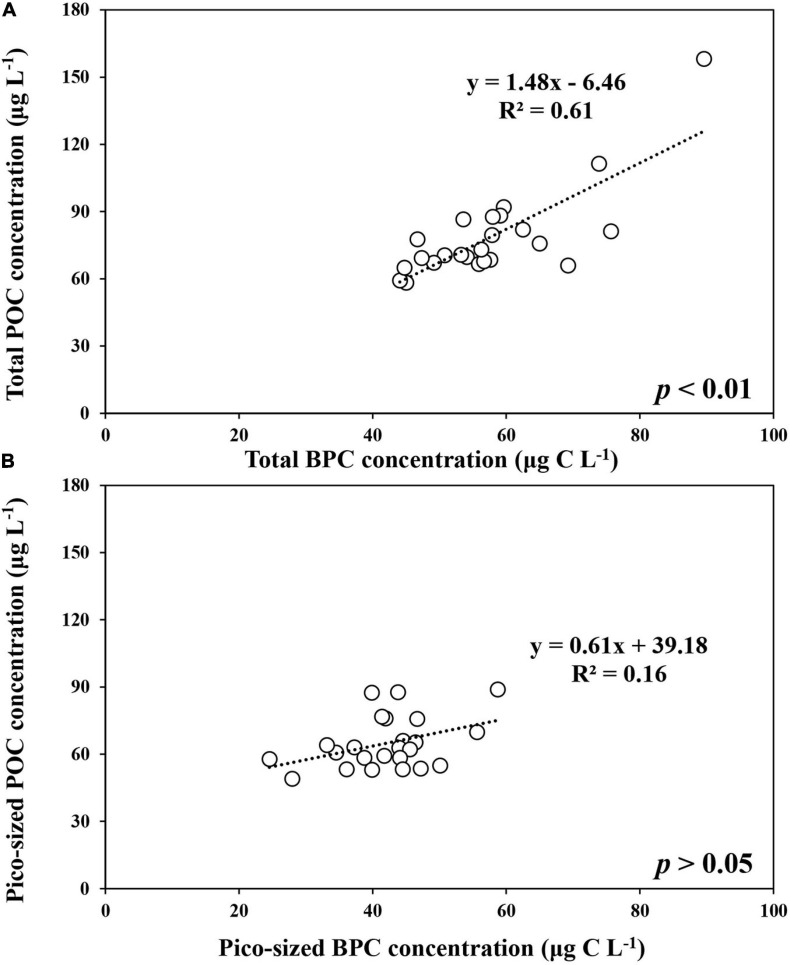
The relationship between POC and BPC concentrations of total (>0.7 μm) **(A)** and pico-sized (0.7–2 μm) **(B)** POM at JBS during the ice-covered period, 2015.

The BPC concentration of total POM ranged from 99.4 to 310.4 μg C L^–1^ with an average of 202.8 μg C L^–1^ (SD = ± 105.6 μg C L^–1^) during the ice-free period (Supplementary Table 1). In comparison, relatively lower concentrations were observed in the ice-covered period, with an average of 57.7 μg C L^–1^ (SD = ± 10.9 μg C L^–1^) (Supplementary Table 1). A strong linear relationship was found between total POC and total BPC concentrations in our study (POC = 1.48^∗^BPC *−*6.46, *r*^2^ = 0.61, *p* < 0.01) (Figure 4A). Compared to the total POM, the estimated BPC concentrations for pico-sized POM were relatively low, with a small variation during the ice-free period (mean ± SD = 68.6 ± 11.9 μg C L^–1^) (Supplementary Table 2). The average BPC concentration for pico-sized POM was 42.0 μg C L^–1^ (SD = ± 7.6 μg C L^–1^), and no significant relationship was found in concentrations between pico-sized POC and pico-sized BPC concentrations during the ice-covered period (Figure 4B).

### Chlorophyll *a* Concentration of Phytoplankton at JBS in 2015

**FIGURE 5:**
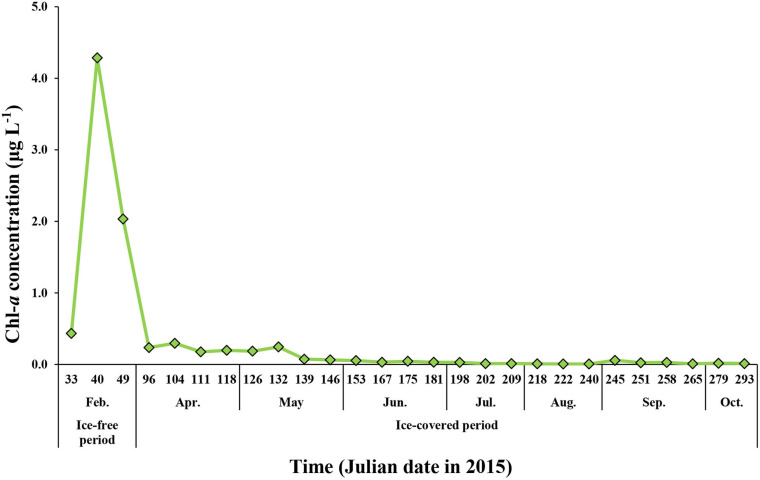
The Chl-*a* concentration for total (>0.7 μm) phytoplankton at JBS during the entire study period, 2015.

The total chlorophyll *a* (Chl-*a*) concentrations of phytoplankton ranged from 0.01 to 4.29 μg L^–1^ (mean ± SD = 0.32 ± 0.88 μg L^–1^) during the whole study period (Figure 5). The maximum concentration was 4.29 μg L^–1,^ which was observed in February when the entire sea ice retreated and the coastal area was opened. The average total Chl-*a* concentrations during the ice-free and ice-covered periods were 2.25 ± 1.93 and 0.08 ± 0.09 μg L^–1^, respectively. Although the average total Chl-*a* concentrations varied considerably between the ice-free period and ice-covered period, there was no statistically significant difference between the two periods (*t*-test, *p* > 0.05) because of a large variation in the Chl-*a* concentration during the ice-free period in this study (Figure 5).

**FIGURE 6:**
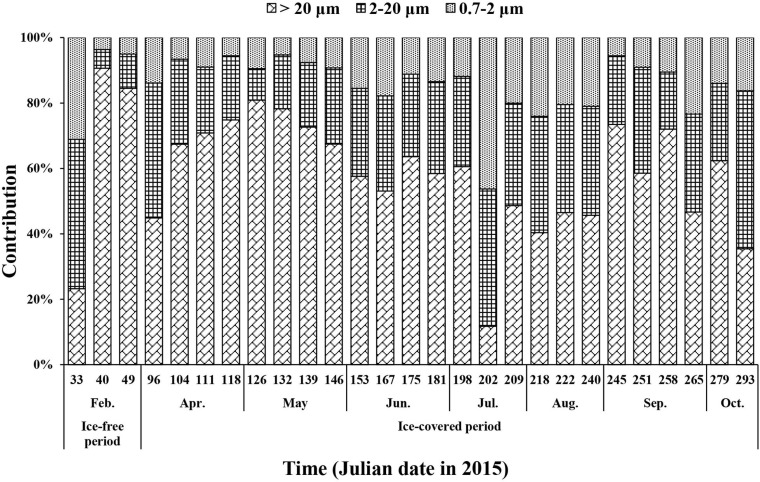
The contributions of size-fractionated Chl-*a* concentration to the total Chl-*a* concentration of phytoplankton at JBS during the entire study period, 2015.

Micro-sized (>20 μm) phytoplankton accounted for up to 91% of the total Chl-*a* during the ice-free period (Figure 6). The average contributions of micro-sized cells to the total Chl-*a* concentration during the ice-free and ice-covered periods were 66 ± 37 and 58 ± 16%, respectively. The contributions of nano- (2–20 μm) and pico-sized (0.7–2 μm) cells were 21 ± 22 and 13 ± 15% during the ice-free period and 28 ± 9 and 14 ± 9% during the ice-covered period, respectively. In other words, micro-sized cells accounted for the largest fraction of Chl-*a* in both periods. Pico-sized cells contributed the least in February (4%) when the micro-sized cells were predominant.

### Macromolecular Composition of the Phytoplankton Community During the Ice-Free and Ice-Covered Periods at JBS in 2015

**FIGURE 7:**
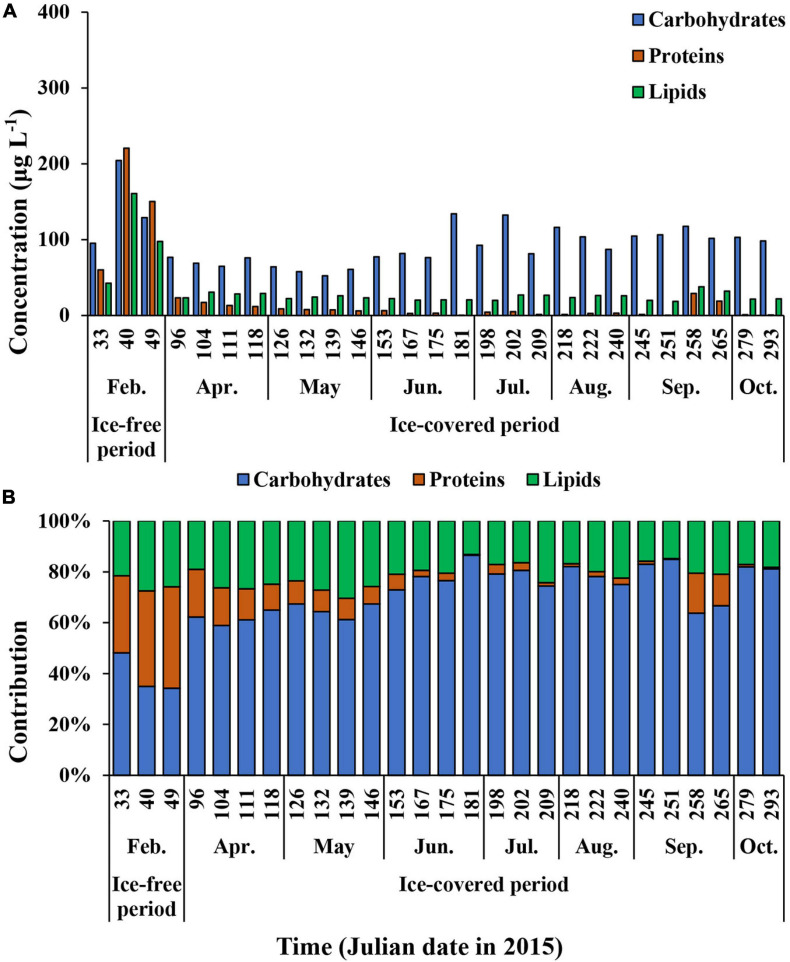
The carbohydrates, proteins, and lipids concentrations **(A)** and the contribution of each biochemical component of total (>0.7 μm) POM **(B)** at JBS during the entire study period, 2015.

**FIGURE 8:**
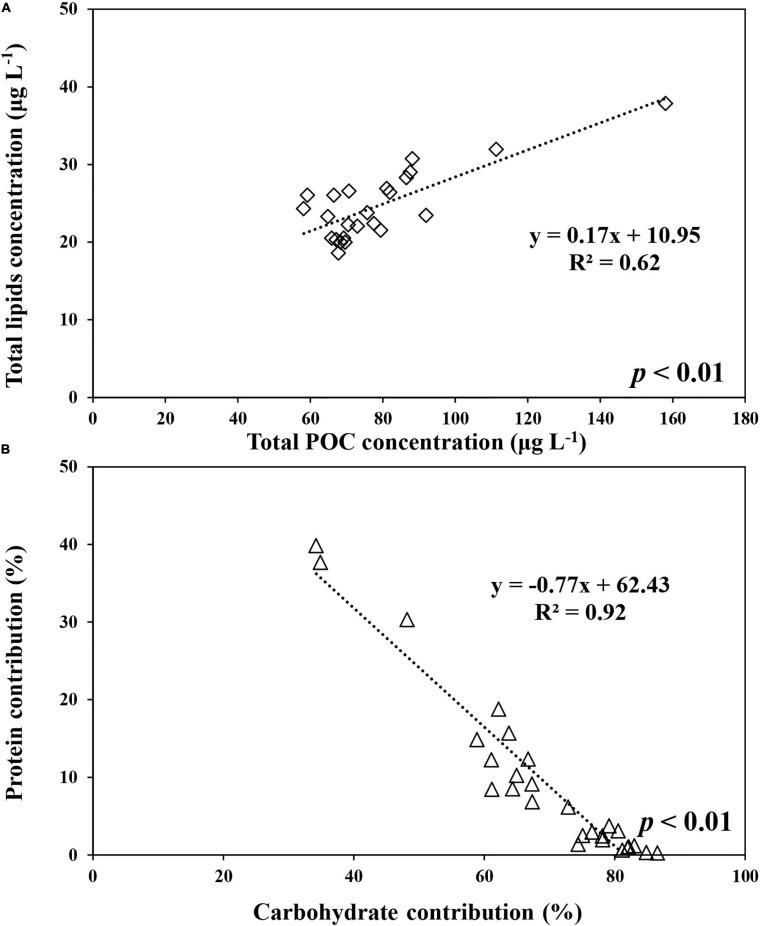
The relationship between lipids and POC concentrations in total (>0.7 μm) POM during the ice-covered period **(A)** and between proteins and carbohydrates contributions to total POM at JBS during the entire study period **(B)**, 2015.

The average carbohydrate, protein, and lipid concentrations in the total POM during the ice-free period were 142.9 ± 55.9, 143.6 ± 80.5, and 100.3 ± 59.1 μg L^–1^, respectively (Figure 7A and Supplementary Table 1). In comparison, the average carbohydrate, protein, and lipid concentrations in the total POM during the ice-covered period were 89.0 ± 23.0, 7.4 ± 7.8, and 24.7 ± 4.6 μg L^–1^, respectively. Each biochemical component was present at higher concentrations during the ice-free period. Among different biochemical components, carbohydrates contributed the most to the total POM (mean ± SD = 69 ± 14%) throughout the entire sampling period, except for on 9 and 18 February, when protein contributed the most (38 and 40% for proteins on each date, respectively) (Figure 7B). The average contribution of carbohydrates to total POM was significantly higher during the ice-covered period, whereas protein contribution was significantly higher during the ice-free period (*t*-test, *p* < 0.05). As the protein concentration decreased more sharply than other macromolecules after the ice-free period, whereas the protein contribution was rapidly decreased over time as well. Similarly, higher lipid concentrations were observed in February, and the concentrations decreased rapidly toward the ice-covered period. In contrast to proteins, lipids were not consumed entirely during the dark winter period, and the average concentration and contribution to the total POM were 24.7 μg L^–1^ (SD = ± 4.6 μg L^–1^) and 21% (SD = ± 4%), respectively. During the ice-covered period, lipid concentrations showed a strong linear relationship with POC (Figure 8A). Overall, the contributions of protein and carbohydrates to the total POM showed a strong negative relationship (*r*^2^ = 0.92, *p* < 0.01) during the entire study period (Figure 8B).

**FIGURE 9:**
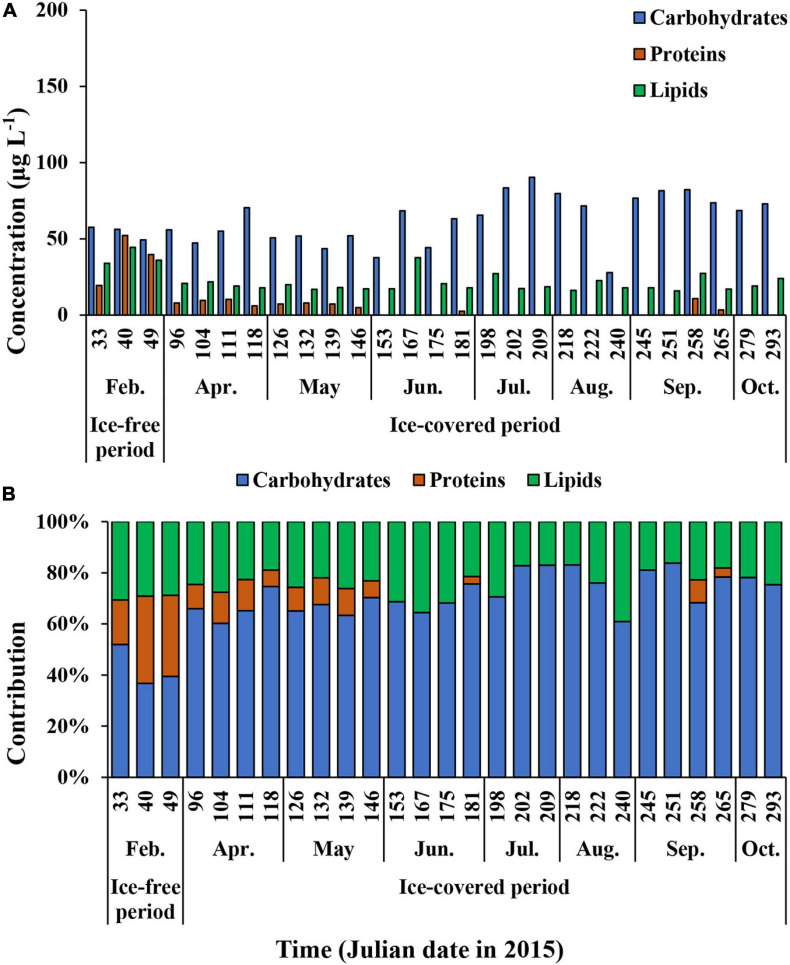
The carbohydrates, proteins, and lipids concentrations **(A)** and the contribution of each biochemical component of pico-sized (0.7–2 μm) POM **(B)** at JBS during the entire study period, 2015.

The carbohydrate, protein, and lipid concentrations in pico-sized (0.7–2 μm) POM during the ice-free period were 54.4 ± 4.4, 37.1 ± 16.6, and 38.1 ± 5.5 μg L^–1^, respectively (Figure 9A and Supplementary Table 2). In comparison, the average concentrations of carbohydrates, proteins, and lipids during the ice-covered period were 63.1 ± 16.3, 3.2 ± 4.0, and 20.3 ± 4.9 μg L^–1^, respectively. Similar to the total POM, the dominant biochemical component of pico-sized POM was carbohydrates (mean ± SD = 69 ± 12%) throughout the entire study period (Figure 9B). Protein concentrations of pico-sized POM were higher in February and decreased sharply over time. However, lipid concentrations and contributions (22.2 ± 7.5 μg L^–1^ and 25 ± 6%, respectively) showed relatively small seasonal variations in pico-sized POM compared to other components during the entire study period.

### Food Materials and Energy Content of Phytoplankton

The average FM (carbohydrates + lipids + proteins) concentration, the calorific value of FM, and calorific content of FM in total POM during the ice-free period were 386.9 μg L^–1^ (SD = ± 194.2 μg L^–1^), 6.0 Kcal g^–1^ (SD = ± 0.2 Kcal g^–1^), and 2.3 Kcal m^–3^ (SD = ± 1.2 Kcal m^–3^), respectively (Supplementary Table 1). During the ice-covered period, the average values for each parameter were 121.1 μg L^–1^ (SD = ± 24.6 μg L^–1^), 5.3 Kcal g^–1^ (SD = ± 0.3 Kcal g^–1^), and 0.6 Kcal m^–3^ (SD = ± 0.1 Kcal m^–3^), respectively (Supplementary Table 1).

The average FM concentration, the calorific value of FM, and the calorific content of FM in pico-sized POM during the ice-free period were 129.7 μg L^–1^ (SD = ± 21.3 μg L^–1^), 6.9 Kcal g^–1^ (SD = ± 0.1 Kcal g^–1^), and 0.8 Kcal m^–3^ (SD = ± 0.1 Kcal m^–3^), respectively (Supplementary Table 2). During the ice-covered period, the average values for each parameter were 86.6 μg L^–1^ (SD = ± 17.0 μg L^–1^), 5.5 Kcal g^–1^ (SD = ± 0.3 Kcal g^–1^), and 0.5 Kcal m^–3^ (SD = ± 0.1 Kcal m^–3^), respectively (Supplementary Table 2).

## Discussion

### Origin of Particulate Organic Matter and Chl-*a* Concentration at JBS in 2015

Seawater is a mixture of various particulate matters, including phytoplankton as well as terrestrial materials. In general, it is well known that the δ^13^C value and C/N ratio provide important information on the origin of organic matter in the oceans (Lobbes et al., 2000; Lee and Whitledge, 2005; Kim et al., 2016). Based on previous results from various oceans (Wada et al., 1987; Zweifel et al., 1993; Fagerbakke et al., 1996; Lobbes et al., 2000; Lee and Whitledge, 2005; Lee et al., 2012; Martiny et al., 2014; Kim et al., 2016), the mean δ^13^C value and C/N ratio obtained during this study indicate that POM was mainly from pelagic phytoplankton. In addition, the BPC contribution to the total POC can vary from 40 to 80% depending on the origin of POM and decrease with increasing terrestrial input (Pusceddu et al., 1996; Fabiano et al., 1997; Danovaro et al., 2000). In this study, the average contribution of BPC to POC (mean ± SD = 74 ± 10%) and the strong positive relationship between BPC and POC during the overall observation period (Figure 4A) suggest that the major source of POM in our study was arguably of oceanic origin. This is not a surprising result given the little terrigenous inputs from Antarctica to the Southern Ocean.

We observed a wide range of total Chl-*a* concentrations (0.01–4.29 μg L^–1^) from February to October in the TNB in 2015. The total Chl-*a* range during the ice-free period in this study is consistent with previous results from the Italian Antarctic Base (Zucchelli Station), which is located at a distance of 10 km from the JBS (Fabiano et al., 1997; Lazzara et al., 1997; Misic et al., 2006) and TNB (Rivaro et al., 2012; Mangoni et al., 2017). During the polar night period, the total Chl-*a* concentrations decreased to 0.01 μg L^–1^ in our current study, which is consistent with the results from McMinn et al. (2010), which ranged from 0.01 to 0.02 μg L^–1^ at McMurdo Sound. These low Chl-*a* concentrations observed in our study site and McMurdo Sound in the Antarctic Ocean are comparable with the results from the Arctic polar night season (Iversen and Seuthe, 2011; Berge et al., 2015).

### Macromolecular Composition During the Ice-Free and Ice-Covered Periods at JBS

The biochemical composition of each constituent in total and pico-sized POM showed a marked difference between the ice-free and ice-covered periods. All components were present at the highest concentrations in February, while they were at the lowest during the ice-covered period. Carbohydrates and lipids were not completely consumed and remained relatively constant, whereas proteins were rarely detected during the ice-covered period. The reason for the maximum concentrations of different macromolecular components during the ice-free period can be explained by the increased light exposure of the phytoplankton community in the surface water layer. Factors controlling the biochemical compositions of phytoplankton in the ocean are light (Fiala and Oriol, 1990; Suárez and Marañón, 2003; Lee et al., 2009), water temperature (Pirt, 1975; Fiala and Oriol, 1990; Kakinuma et al., 2006; Doney et al., 2012), macro-nutrients (Fabiano et al., 1993; Biddanda and Benner, 1997; Lee et al., 2009; Kim et al., 2015), and micro-nutrients availability (Sedwick et al., 2000, 2011; Zhu et al., 2016). Favorable light conditions can increase the growth rate of phytoplankton (Fiala and Oriol, 1990). Excessive light intensity, however, can decrease the protein content of phytoplankton (Suárez and Marañón, 2003; Lee et al., 2008, 2009), whereas insufficient light can increase the carbohydrate and lipid composition in phytoplankton (Friedman et al., 1991; Suárez and Marañón, 2003). Low water temperatures can reduce the metabolic rate and growth rate of phytoplankton (Fiala and Oriol, 1990). In a nitrogen-rich environment, phytoplankton can actively accumulate proteins in the cell body during photosynthesis (Fabiano et al., 1993; Lee et al., 2009), whereas in the opposite environments, lipid and carbohydrate synthesis predominates (Shifrin and Chisholm, 1981; Harrison et al., 1990).

Among the environmental conditions affecting the primary productivity and biochemical composition of phytoplankton, light undergoes the most dramatic seasonal changes depending on sea ice conditions. Our study area was covered with land-fast sea ice before February and opened during February. As mentioned above, extreme light intensity could limit the protein synthesis of phytoplankton (Suárez and Marañón, 2003; Lee et al., 2008, 2009). However, the increased protein content observed in February indicates that phytoplankton did not experience strong light inhibition during the growing period. The protein:carbohydrate ratios could reflect the nutrient availability and productivity for phytoplankton (Fabiano et al., 1984, 1992, 1993; Mayzaud et al., 1989; Lizotte and Sullivan, 1992; Danovaro et al., 2000). A ratio less than 1 indicates nitrogen deficiency for phytoplankton growth (Mayzaud et al., 1989; Lizotte and Sullivan, 1992; Danovaro et al., 2000; Lee et al., 2009; Yun et al., 2015), whereas a ratio higher than 1 could be observed in productive areas or phytoplankton bloom periods (Fabiano et al., 1984, 1992, 1993; Lee et al., 2009). The average protein:carbohydrate ratio was 1.0 ± 0.3 during the ice-free period, which suggests that no nitrogen limitation occurred during this period in February. Previous studies demonstrate that nitrogen limitation is typically not observed in the Southern Ocean (de Baar et al., 1995; Boyd et al., 2000; Franck et al., 2000; Henley et al., 2020). Unfortunately, we did not measure macronutrient concentrations to confirm this.

In the ice-covered period, the phytoplankton community undergoes extremely low light conditions because of sea ice cover as well as polar night. Handa (1969) reported a change in the biochemical composition of the marine diatom *Skeletonema costatum* after 18 days of incubation under dark conditions. At the beginning of the dark condition, *Skeletonema costatum* used readily available non-structural carbohydrates, glucose, and β-1,3-glucan, while cell-structural carbohydrates such as mannan and pentosan were not used for respiration (Handa, 1969). After consuming non-structural carbohydrates, they consume proteins for survival. In contrast to carbohydrates and proteins, no significant fluctuations in lipids were observed. Smayda and Mitchell-Innes (1974) reported how long *Skeletonema costatum* could survive in dark conditions. Interestingly, they survived only 1–4 weeks at 20°C under dark conditions, but they survived 24 weeks at 2°C without light. In addition, Bunt and Lee (1972) reported that two diatoms isolated from Antarctic sea ice also survived for 90 days (duration of the experiment) of darkness at −1.8°C. This suggests that low temperatures under dark conditions can play an important role in the survival of diatoms. In our study, the average water temperature during the ice-covered periods was −1.78°C (SD = ± 0.04°C) in 2015. In addition, the coastal area of the TNB is well known to be dominated with diatoms (Arrigo et al., 2003; Fonda Umani et al., 2005; Mangoni et al., 2019).

Many types of phytoplankton are known to experience a resting stage in their life cycle (Ellegaard and Ribeiro, 2018). Some resting stages are related to sexual reproduction, while other resting stages are asexual and form solely following changes in environmental conditions toward the end of the growing season, such as akinetes in some cyanobacteria and resting spores in some diatoms (Ellegaard and Ribeiro, 2018). Diatom resting spores have morphological characteristics distinct from those of fresh living cells, and they are more heavily silicified (Oku and Kamatani, 1995; McQuoid and Hobson, 1996). In addition, they are known to accumulate carbohydrates and lipids as energy storage materials in resting spores, and they accumulate large amounts of organic carbon in the form of neutral lipids (Oku and Kamatani, 1999). In this study, a strong positive relationship between lipid concentrations and total POC during the ice-covered period (Figure 9A) suggests that phytoplankton survived with lipids as their energy source during the long cold darkness in our observation period.

The average protein:carbohydrate ratio decreased markedly during the ice-covered period (mean ± SD = 0.1 ± 0.1) compared to the ice-free period (mean ± SD = 1.0 ± 0.3). This observation may be explained by the difference in the physiological state of phytoplankton during the two periods. In the ice-free period, phytoplankton could utilize sufficient photosynthetically available radiation and nutrients, and as a result, the overall macromolecules in the cell body were increased, with a particularly marked increase in proteins. During the ice-covered period, however, the active growing season was over due to the formation of sea ice. Phytoplankton might consume immediately available forms of carbohydrates such as glucose as a strategy for survival followed by most of the proteins. Therefore, the physiological conditions of phytoplankton in the two different periods could cause a negative relationship between the protein and carbohydrate contributions to the total POM in our study. Another possibility for different macromolecular compositions between the ice-free and ice-covered periods could be a change in dominant phytoplankton groups that affects the biochemical composition of POM (Moal et al., 1987; Rivkin and Voytek, 1987; Harrison et al., 1990; Kim et al., 2018). Kim et al. (2018) found that high carbohydrate compositions were caused by the enhanced contribution of *P. Antarctica* in the Amundsen Sea. However, we did not verify the potential effects of the shift in the species makeup on the macromolecular compositions because we did not investigate species breakdown of phytoplankton in this study. Generally speaking, diatoms are reported to be dominant in the phytoplankton community in the TNB (Arrigo et al., 2003; Fonda Umani et al., 2005; Mangoni et al., 2019). In particular, at JBS in the TNB, the phytoplankton community was predominated by diatoms during summer (85.3%) and during fall and winter periods (>95%) in 2018 (unpublished data). Therefore, the change in major species compositions of phytoplankton is less likely to account for the difference in macromolecular compositions between the ice-free and ice-covered periods.

Among the three biochemical components, proteins decreased most rapidly with time and were low from April to October 2015. The substantial decrease in protein concentrations observed in this study is similar to the composition change in sinking particles from the euphotic layer to the aphotic layer in the Amundsen Sea reported by Kim et al. (2018). Previous studies reported that proteins consisted of various amino acids that could be consumed more easily than other compounds (Handa and Tominaga, 1969; Dawson and Liebezeit, 1982; Fabiano et al., 1995; Hedges et al., 2001; Danovaro et al., 2000). In addition, several previous studies reported that the carbohydrates in sinking particles underwent limited decomposition compared to the other components (Ittekkot et al., 1982; Liebezeit, 1984; Fabiano et al., 1993; Danovaro et al., 2000; Kim et al., 2018). Approximately 5% of proteins and 60% of carbohydrates produced during the ice-free period remained during the ice-covered period. Compared to carbohydrates and proteins, relatively higher fraction of lipids (25%) remained during the ice-covered period. Lipids might play an important role in the survival of phytoplankton, lowering the metabolic rate during the long ice-covered period. Bunt et al. (1966) reported that lower water temperatures reduced the metabolism of *Fragilaria sublinearis* and that at 3°C dark respiration decreased to less than 50% of that at 10°C. Palmisano and Sullivan, 1982 reported that diatoms could use stored energy products to survive under lowered metabolic activity at low temperatures. The use of carbohydrates and lipids in the cell body might be a critical survival strategy for phytoplankton during the long ice-covered polar night.

### Food Materials and Energy Content of Phytoplankton

Previous studies reported high FM concentrations in the euphotic zone in the Southern Ocean with a large proportion of proteins (Fabiano et al., 1993, 1996; Kim et al., 2016) although Kim et al. (2018) observed carbohydrate-dominant POM in the *P. antarctica*-prevalent community of the Amundsen Sea. While the POM in the euphotic layer sinks to the aphotic layer upon decomposition, the relative contribution of carbohydrates could increase due to the selective degradation of proteins by heterotrophs such as bacteria (Fabiano et al., 1993, 1996; Kim et al., 2016, 2018). The average calorific contents of FM were 2.3 ± 1.2 and 0.6 ± 0.1 Kcal m^–3^ for the ice-free and ice-covered periods, respectively. These values fall in the range reported from the Southern Ocean (Supplementary Table 3). Generally, the calorific content of FM has a very low value in the aphotic layer compared to that in the photic layer due to a decrease in the overall FM concentration as well as an increasing contribution of carbohydrates, which has the smallest energy content per unit weight among the three different types of macromolecules (Fabiano et al., 1993, 1996; Kim et al., 2016, 2018). Similar to previous studies reporting the high biochemical components in the euphotic zone (Fabiano et al., 1993, 1996; Kim et al., 2016, 2018), we found the highest FM concentrations (585.8 μg L^–1^) as well as the calorific content of FM (3.6 Kcal m^–3^) in the surface layer at JBS during the active growth phase in the ice-free period. In this period, the protein concentration was the highest among the different biochemical components. After that, the protein concentrations decreased rapidly over time, and the FM concentration and calorific content of FM had minimum values (85.8 μg L^–1^ and 0.5 Kcal m^–3^ for the minimum FM concentration and calorific content of FM, respectively) during the ice-covered period.

Previous studies reported that the dominant phytoplankton size might change to pico-sized cells due to the warming ocean (Li et al., 2009; Morán et al., 2010). To assess the relative contribution of the pico-sized cell to the total POM in the current study, we calculated the ratios (pico-size:total) of Chl-*a*, FM, and calorific content of FM based on the cell size of POM. During the ice-free period, the ratio of Chl-*a* was 0.13 ± 0.16, and the ratios of FM and calorific content were 0.38 ± 0.16 and 0.40 ± 0.17, respectively. In addition, the ratio of Chl-*a* was 0.15 ± 0.10, and the ratios of FM and calorific content were 0.73 ± 0.14 and 0.74 ± 0.14 during the ice-covered period, respectively. The contribution of pico-sized Chl-*a* to the total Chl-*a* was lower than those of FM and calorific content during the entire study period (*t*-test, *p* < 0.05). Moreover, the FM and the calorific content per unit of Chl-*a* of pico-sized POM were higher than those of the total POM (*t*-test, *p* < 0.05). This suggests that the pico-sized cells could accumulate FM and energy per unit Chl-*a* into the cell body more efficiently than larger cells. Consistent with our findings, Kang et al. (2017) observed higher FM and calorific content per unit Chl-*a* of small phytoplankton (0.7–2 μm) compared to the total phytoplankton in the East Sea.

## Summary and Conclusion

This study reported on the biweekly variations in biomass and physiological state of phytoplankton from the JBS located in the coastal region of the TNB in the Ross Sea from the ice-free growing period to the ice-covered polar night. During the ice-free period, we observed a high Chl-*a* concentration with a high proportion of micro-sized cells for the whole phytoplankton community. Each macromolecular component in phytoplankton showed maximum values during the ice-free period. Interestingly, a relatively higher protein contribution was found compared to carbohydrates and lipids in this period. After the study area was covered with sea ice in early March, the Chl-*a* concentration decreased sharply, and the concentration of macromolecules in phytoplankton also decreased, but each component showed different patterns. Carbohydrates showed only a small decrease compared to the other two constituents and consequently became a major component of POM during the dark winter period. Similar to the pattern of Chl-*a*, the protein concentration decreased rapidly over time and was close to detection levels during the ice-covered period, while the lipids were not consumed completely. The proteins appear to be consumed favorably, and lipids might be an important energy source for living during the long ice-covered period for phytoplankton.

Earlier studies reported different trends in the sea ice extent in the different regions of the Southern Ocean. Changes in the timing of sea ice melt onset and duration of the ice-free period could influence the growing conditions and consequently biochemical compositions of phytoplankton. Because of the limitation of geographical accessibility, very little information is available on how the biochemical compositions of phytoplankton vary from the ice-free period and ice-covered period. This study could provide valuable basic data for understanding the effect of future climate change on phytoplankton in the Southern Ocean. Continuous monitoring is needed on the macromolecular compositions of phytoplankton as an indicator of climate change in the Southern Ocean, which is facing dramatic environmental changes.

## Data Availability Statement

The original contributions presented in the study are included in the article/Supplementary Material, further inquiries can be directed to the corresponding author/s.

## Author Contributions

SL conceived the ideas and designed the methodology. KK performed the field experiments and data analysis. KK, NJ, SP, HY, and JK conducted the lab experiment. KK and SL contributed to writing–original draft. KK, JP, and SL contributed to writing–review and editing. All authors agreed with the submission of the published version of the manuscript.

## Conflict of Interest

The authors declare that the research was conducted in the absence of any commercial or financial relationships that could be construed as a potential conflict of interest.

## Supplementary Material

Supplementary Table 1 The macromolecules (carbohydrates, proteins, and lipids), POC, PON, BPC concentrations, and associated calorific value of FM for total (> 0.7 μm) POM at the JBS, 2015.

Supplementary Table 2 The macromolecules (carbohydrates, proteins, and lipids), POC, PON, BPC concentrations, and associated calorific value of FM for pico-sized (0.7-2 μm) POM at the JBS, 2015.

Supplementary Table 3 Comparison of FM concentration and calorific content of FM in the Southern Ocean (Ross Sea and Amundsen Sea).
