# Supplementary material for: Monthly Variation in the Macromolecular Composition of Phytoplankton Communities at Jang Bogo Station, Terra Nova Bay, Ross Sea
Source: Front Microbiol. 2021 Feb 11;12:618999. doi: 10.3389/fmicb.2021.618999 (PMC7905043; doi:10.3389/fmicb.2021.618999)
Supplement: Supplementary Table 1 — The macromolecules (carbohydrates, proteins, and lipids), POC, PON, BPC concentrations, and associated calorific value of FM for total (> 0.7 μm) POM at the JBS, 2015. [file Presentation_1.PPTX]

## Slide 1
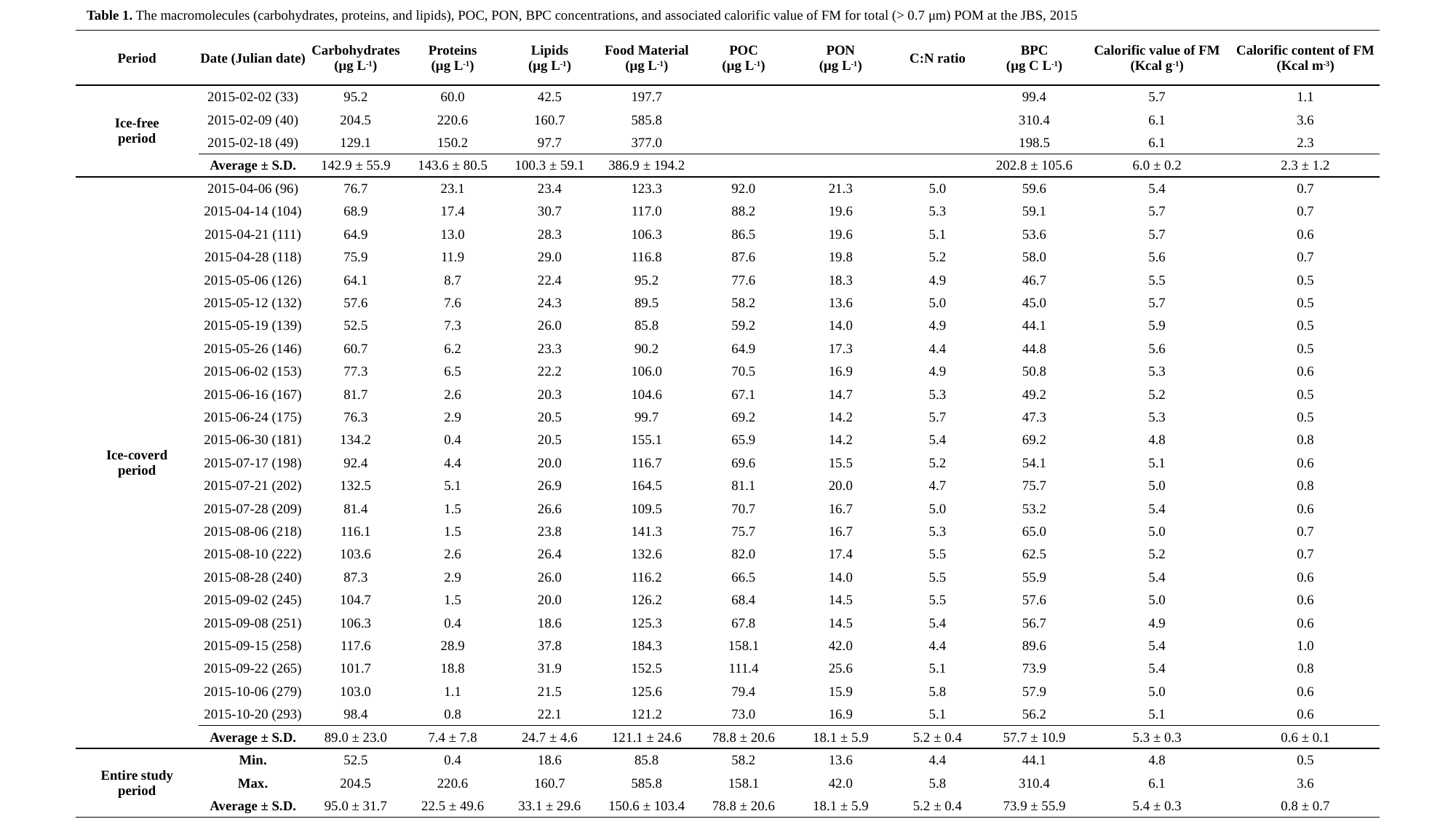

| Table 1. The macromolecules (carbohydrates, proteins, and lipids), POC, PON, BPC concentrations, and associated calorific value of FM for total (> 0.7 μm) POM at the JBS, 2015 | | | | | | | | | | | |
| --- | --- | --- | --- | --- | --- | --- | --- | --- | --- | --- | --- |
| Period | Date (Julian date) | Carbohydrates (μg L-1) | Proteins (μg L-1) | Lipids (μg L-1) | Food Material (μg L-1) | POC (μg L-1) | PON (μg L-1) | C:N ratio | BPC (μg C L-1) | Calorific value of FM (Kcal g-1) | Calorific content of FM (Kcal m-3) |
| Ice-free period | 2015-02-02 (33) | 95.2 | 60.0 | 42.5 | 197.7 | | | | 99.4 | 5.7 | 1.1 |
| | 2015-02-09 (40) | 204.5 | 220.6 | 160.7 | 585.8 | | | | 310.4 | 6.1 | 3.6 |
| | 2015-02-18 (49) | 129.1 | 150.2 | 97.7 | 377.0 | | | | 198.5 | 6.1 | 2.3 |
| | Average ± S.D. | 142.9 ± 55.9 | 143.6 ± 80.5 | 100.3 ± 59.1 | 386.9 ± 194.2 | | | | 202.8 ± 105.6 | 6.0 ± 0.2 | 2.3 ± 1.2 |
| Ice-coverd period | 2015-04-06 (96) | 76.7 | 23.1 | 23.4 | 123.3 | 92.0 | 21.3 | 5.0 | 59.6 | 5.4 | 0.7 |
| | 2015-04-14 (104) | 68.9 | 17.4 | 30.7 | 117.0 | 88.2 | 19.6 | 5.3 | 59.1 | 5.7 | 0.7 |
| | 2015-04-21 (111) | 64.9 | 13.0 | 28.3 | 106.3 | 86.5 | 19.6 | 5.1 | 53.6 | 5.7 | 0.6 |
| | 2015-04-28 (118) | 75.9 | 11.9 | 29.0 | 116.8 | 87.6 | 19.8 | 5.2 | 58.0 | 5.6 | 0.7 |
| | 2015-05-06 (126) | 64.1 | 8.7 | 22.4 | 95.2 | 77.6 | 18.3 | 4.9 | 46.7 | 5.5 | 0.5 |
| | 2015-05-12 (132) | 57.6 | 7.6 | 24.3 | 89.5 | 58.2 | 13.6 | 5.0 | 45.0 | 5.7 | 0.5 |
| | 2015-05-19 (139) | 52.5 | 7.3 | 26.0 | 85.8 | 59.2 | 14.0 | 4.9 | 44.1 | 5.9 | 0.5 |
| | 2015-05-26 (146) | 60.7 | 6.2 | 23.3 | 90.2 | 64.9 | 17.3 | 4.4 | 44.8 | 5.6 | 0.5 |
| | 2015-06-02 (153) | 77.3 | 6.5 | 22.2 | 106.0 | 70.5 | 16.9 | 4.9 | 50.8 | 5.3 | 0.6 |
| | 2015-06-16 (167) | 81.7 | 2.6 | 20.3 | 104.6 | 67.1 | 14.7 | 5.3 | 49.2 | 5.2 | 0.5 |
| | 2015-06-24 (175) | 76.3 | 2.9 | 20.5 | 99.7 | 69.2 | 14.2 | 5.7 | 47.3 | 5.3 | 0.5 |
| | 2015-06-30 (181) | 134.2 | 0.4 | 20.5 | 155.1 | 65.9 | 14.2 | 5.4 | 69.2 | 4.8 | 0.8 |
| | 2015-07-17 (198) | 92.4 | 4.4 | 20.0 | 116.7 | 69.6 | 15.5 | 5.2 | 54.1 | 5.1 | 0.6 |
| | 2015-07-21 (202) | 132.5 | 5.1 | 26.9 | 164.5 | 81.1 | 20.0 | 4.7 | 75.7 | 5.0 | 0.8 |
| | 2015-07-28 (209) | 81.4 | 1.5 | 26.6 | 109.5 | 70.7 | 16.7 | 5.0 | 53.2 | 5.4 | 0.6 |
| | 2015-08-06 (218) | 116.1 | 1.5 | 23.8 | 141.3 | 75.7 | 16.7 | 5.3 | 65.0 | 5.0 | 0.7 |
| | 2015-08-10 (222) | 103.6 | 2.6 | 26.4 | 132.6 | 82.0 | 17.4 | 5.5 | 62.5 | 5.2 | 0.7 |
| | 2015-08-28 (240) | 87.3 | 2.9 | 26.0 | 116.2 | 66.5 | 14.0 | 5.5 | 55.9 | 5.4 | 0.6 |
| | 2015-09-02 (245) | 104.7 | 1.5 | 20.0 | 126.2 | 68.4 | 14.5 | 5.5 | 57.6 | 5.0 | 0.6 |
| | 2015-09-08 (251) | 106.3 | 0.4 | 18.6 | 125.3 | 67.8 | 14.5 | 5.4 | 56.7 | 4.9 | 0.6 |
| | 2015-09-15 (258) | 117.6 | 28.9 | 37.8 | 184.3 | 158.1 | 42.0 | 4.4 | 89.6 | 5.4 | 1.0 |
| | 2015-09-22 (265) | 101.7 | 18.8 | 31.9 | 152.5 | 111.4 | 25.6 | 5.1 | 73.9 | 5.4 | 0.8 |
| | 2015-10-06 (279) | 103.0 | 1.1 | 21.5 | 125.6 | 79.4 | 15.9 | 5.8 | 57.9 | 5.0 | 0.6 |
| | 2015-10-20 (293) | 98.4 | 0.8 | 22.1 | 121.2 | 73.0 | 16.9 | 5.1 | 56.2 | 5.1 | 0.6 |
| | Average ± S.D. | 89.0 ± 23.0 | 7.4 ± 7.8 | 24.7 ± 4.6 | 121.1 ± 24.6 | 78.8 ± 20.6 | 18.1 ± 5.9 | 5.2 ± 0.4 | 57.7 ± 10.9 | 5.3 ± 0.3 | 0.6 ± 0.1 |
| Entire study period | Min. | 52.5 | 0.4 | 18.6 | 85.8 | 58.2 | 13.6 | 4.4 | 44.1 | 4.8 | 0.5 |
| | Max. | 204.5 | 220.6 | 160.7 | 585.8 | 158.1 | 42.0 | 5.8 | 310.4 | 6.1 | 3.6 |
| | Average ± S.D. | 95.0 ± 31.7 | 22.5 ± 49.6 | 33.1 ± 29.6 | 150.6 ± 103.4 | 78.8 ± 20.6 | 18.1 ± 5.9 | 5.2 ± 0.4 | 73.9 ± 55.9 | 5.4 ± 0.3 | 0.8 ± 0.7 |
