# Supplementary material for: Monthly Variation in the Macromolecular Composition of Phytoplankton Communities at Jang Bogo Station, Terra Nova Bay, Ross Sea
Source: Front Microbiol. 2021 Feb 11;12:618999. doi: 10.3389/fmicb.2021.618999 (PMC7905043; doi:10.3389/fmicb.2021.618999)
Supplement: Supplementary Table 2 — The macromolecules (carbohydrates, proteins, and lipids), POC, PON, BPC concentrations, and associated calorific value of FM for pico-sized (0.7-2 μm) POM at the JBS, 2015. [file Presentation_2.PPTX]

## Slide 1
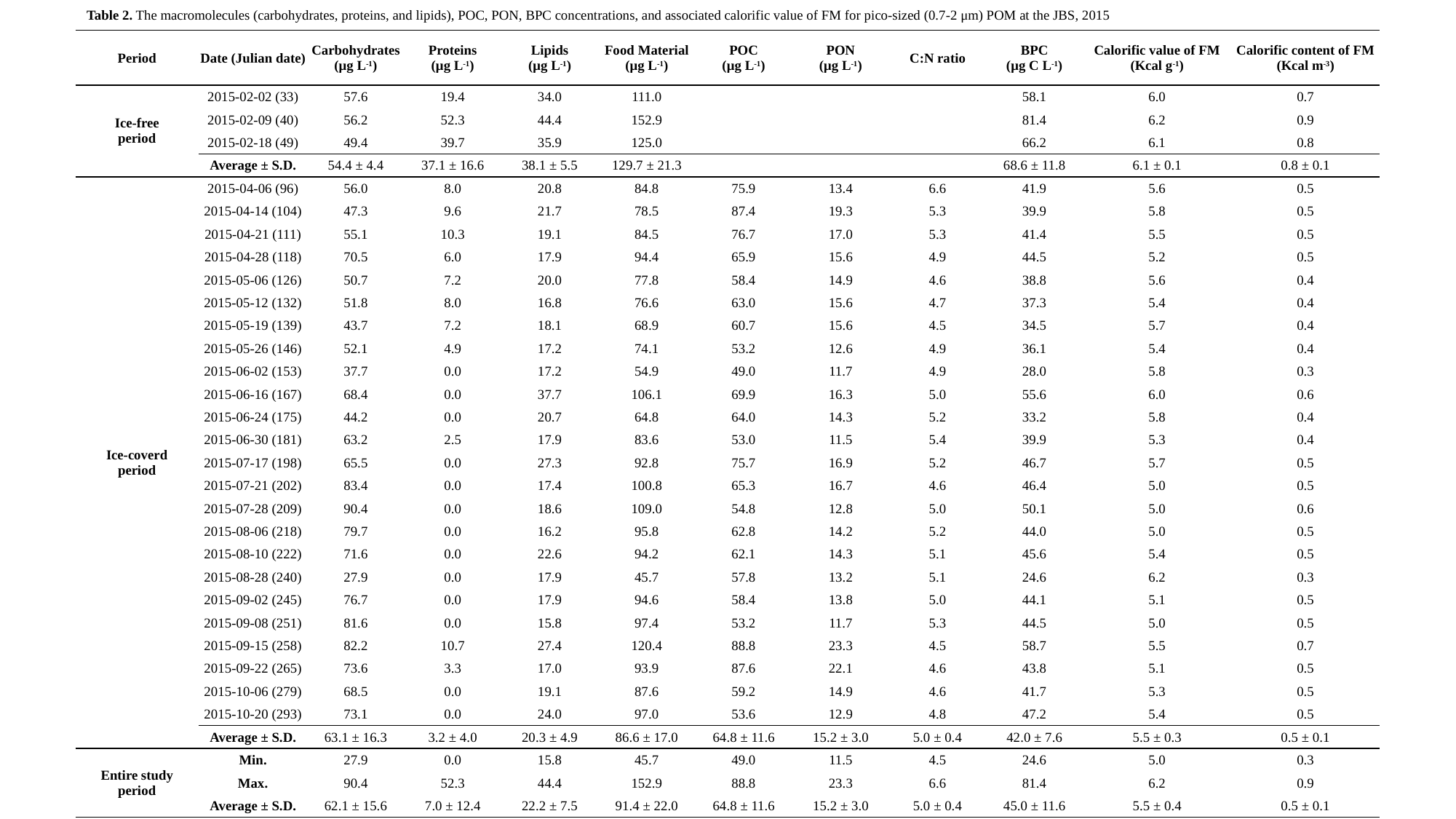

| Table 2. The macromolecules (carbohydrates, proteins, and lipids), POC, PON, BPC concentrations, and associated calorific value of FM for pico-sized (0.7-2 μm) POM at the JBS, 2015 | | | | | | | | | | | |
| --- | --- | --- | --- | --- | --- | --- | --- | --- | --- | --- | --- |
| Period | Date (Julian date) | Carbohydrates (μg L-1) | Proteins (μg L-1) | Lipids (μg L-1) | Food Material (μg L-1) | POC (μg L-1) | PON (μg L-1) | C:N ratio | BPC (μg C L-1) | Calorific value of FM (Kcal g-1) | Calorific content of FM (Kcal m-3) |
| Ice-free period | 2015-02-02 (33) | 57.6 | 19.4 | 34.0 | 111.0 | | | | 58.1 | 6.0 | 0.7 |
| | 2015-02-09 (40) | 56.2 | 52.3 | 44.4 | 152.9 | | | | 81.4 | 6.2 | 0.9 |
| | 2015-02-18 (49) | 49.4 | 39.7 | 35.9 | 125.0 | | | | 66.2 | 6.1 | 0.8 |
| | Average ± S.D. | 54.4 ± 4.4 | 37.1 ± 16.6 | 38.1 ± 5.5 | 129.7 ± 21.3 | | | | 68.6 ± 11.8 | 6.1 ± 0.1 | 0.8 ± 0.1 |
| Ice-coverd period | 2015-04-06 (96) | 56.0 | 8.0 | 20.8 | 84.8 | 75.9 | 13.4 | 6.6 | 41.9 | 5.6 | 0.5 |
| | 2015-04-14 (104) | 47.3 | 9.6 | 21.7 | 78.5 | 87.4 | 19.3 | 5.3 | 39.9 | 5.8 | 0.5 |
| | 2015-04-21 (111) | 55.1 | 10.3 | 19.1 | 84.5 | 76.7 | 17.0 | 5.3 | 41.4 | 5.5 | 0.5 |
| | 2015-04-28 (118) | 70.5 | 6.0 | 17.9 | 94.4 | 65.9 | 15.6 | 4.9 | 44.5 | 5.2 | 0.5 |
| | 2015-05-06 (126) | 50.7 | 7.2 | 20.0 | 77.8 | 58.4 | 14.9 | 4.6 | 38.8 | 5.6 | 0.4 |
| | 2015-05-12 (132) | 51.8 | 8.0 | 16.8 | 76.6 | 63.0 | 15.6 | 4.7 | 37.3 | 5.4 | 0.4 |
| | 2015-05-19 (139) | 43.7 | 7.2 | 18.1 | 68.9 | 60.7 | 15.6 | 4.5 | 34.5 | 5.7 | 0.4 |
| | 2015-05-26 (146) | 52.1 | 4.9 | 17.2 | 74.1 | 53.2 | 12.6 | 4.9 | 36.1 | 5.4 | 0.4 |
| | 2015-06-02 (153) | 37.7 | 0.0 | 17.2 | 54.9 | 49.0 | 11.7 | 4.9 | 28.0 | 5.8 | 0.3 |
| | 2015-06-16 (167) | 68.4 | 0.0 | 37.7 | 106.1 | 69.9 | 16.3 | 5.0 | 55.6 | 6.0 | 0.6 |
| | 2015-06-24 (175) | 44.2 | 0.0 | 20.7 | 64.8 | 64.0 | 14.3 | 5.2 | 33.2 | 5.8 | 0.4 |
| | 2015-06-30 (181) | 63.2 | 2.5 | 17.9 | 83.6 | 53.0 | 11.5 | 5.4 | 39.9 | 5.3 | 0.4 |
| | 2015-07-17 (198) | 65.5 | 0.0 | 27.3 | 92.8 | 75.7 | 16.9 | 5.2 | 46.7 | 5.7 | 0.5 |
| | 2015-07-21 (202) | 83.4 | 0.0 | 17.4 | 100.8 | 65.3 | 16.7 | 4.6 | 46.4 | 5.0 | 0.5 |
| | 2015-07-28 (209) | 90.4 | 0.0 | 18.6 | 109.0 | 54.8 | 12.8 | 5.0 | 50.1 | 5.0 | 0.6 |
| | 2015-08-06 (218) | 79.7 | 0.0 | 16.2 | 95.8 | 62.8 | 14.2 | 5.2 | 44.0 | 5.0 | 0.5 |
| | 2015-08-10 (222) | 71.6 | 0.0 | 22.6 | 94.2 | 62.1 | 14.3 | 5.1 | 45.6 | 5.4 | 0.5 |
| | 2015-08-28 (240) | 27.9 | 0.0 | 17.9 | 45.7 | 57.8 | 13.2 | 5.1 | 24.6 | 6.2 | 0.3 |
| | 2015-09-02 (245) | 76.7 | 0.0 | 17.9 | 94.6 | 58.4 | 13.8 | 5.0 | 44.1 | 5.1 | 0.5 |
| | 2015-09-08 (251) | 81.6 | 0.0 | 15.8 | 97.4 | 53.2 | 11.7 | 5.3 | 44.5 | 5.0 | 0.5 |
| | 2015-09-15 (258) | 82.2 | 10.7 | 27.4 | 120.4 | 88.8 | 23.3 | 4.5 | 58.7 | 5.5 | 0.7 |
| | 2015-09-22 (265) | 73.6 | 3.3 | 17.0 | 93.9 | 87.6 | 22.1 | 4.6 | 43.8 | 5.1 | 0.5 |
| | 2015-10-06 (279) | 68.5 | 0.0 | 19.1 | 87.6 | 59.2 | 14.9 | 4.6 | 41.7 | 5.3 | 0.5 |
| | 2015-10-20 (293) | 73.1 | 0.0 | 24.0 | 97.0 | 53.6 | 12.9 | 4.8 | 47.2 | 5.4 | 0.5 |
| | Average ± S.D. | 63.1 ± 16.3 | 3.2 ± 4.0 | 20.3 ± 4.9 | 86.6 ± 17.0 | 64.8 ± 11.6 | 15.2 ± 3.0 | 5.0 ± 0.4 | 42.0 ± 7.6 | 5.5 ± 0.3 | 0.5 ± 0.1 |
| Entire study period | Min. | 27.9 | 0.0 | 15.8 | 45.7 | 49.0 | 11.5 | 4.5 | 24.6 | 5.0 | 0.3 |
| | Max. | 90.4 | 52.3 | 44.4 | 152.9 | 88.8 | 23.3 | 6.6 | 81.4 | 6.2 | 0.9 |
| | Average ± S.D. | 62.1 ± 15.6 | 7.0 ± 12.4 | 22.2 ± 7.5 | 91.4 ± 22.0 | 64.8 ± 11.6 | 15.2 ± 3.0 | 5.0 ± 0.4 | 45.0 ± 11.6 | 5.5 ± 0.4 | 0.5 ± 0.1 |
