# Supplementary material for: Monthly Variation in the Macromolecular Composition of Phytoplankton Communities at Jang Bogo Station, Terra Nova Bay, Ross Sea
Source: Front Microbiol. 2021 Feb 11;12:618999. doi: 10.3389/fmicb.2021.618999 (PMC7905043; doi:10.3389/fmicb.2021.618999)
Supplement: Supplementary Table 3 — Comparison of FM concentration and calorific content of FM in the Southern Ocean (Ross Sea and Amundsen Sea). [file Presentation_3.PPTX]

## Slide 1
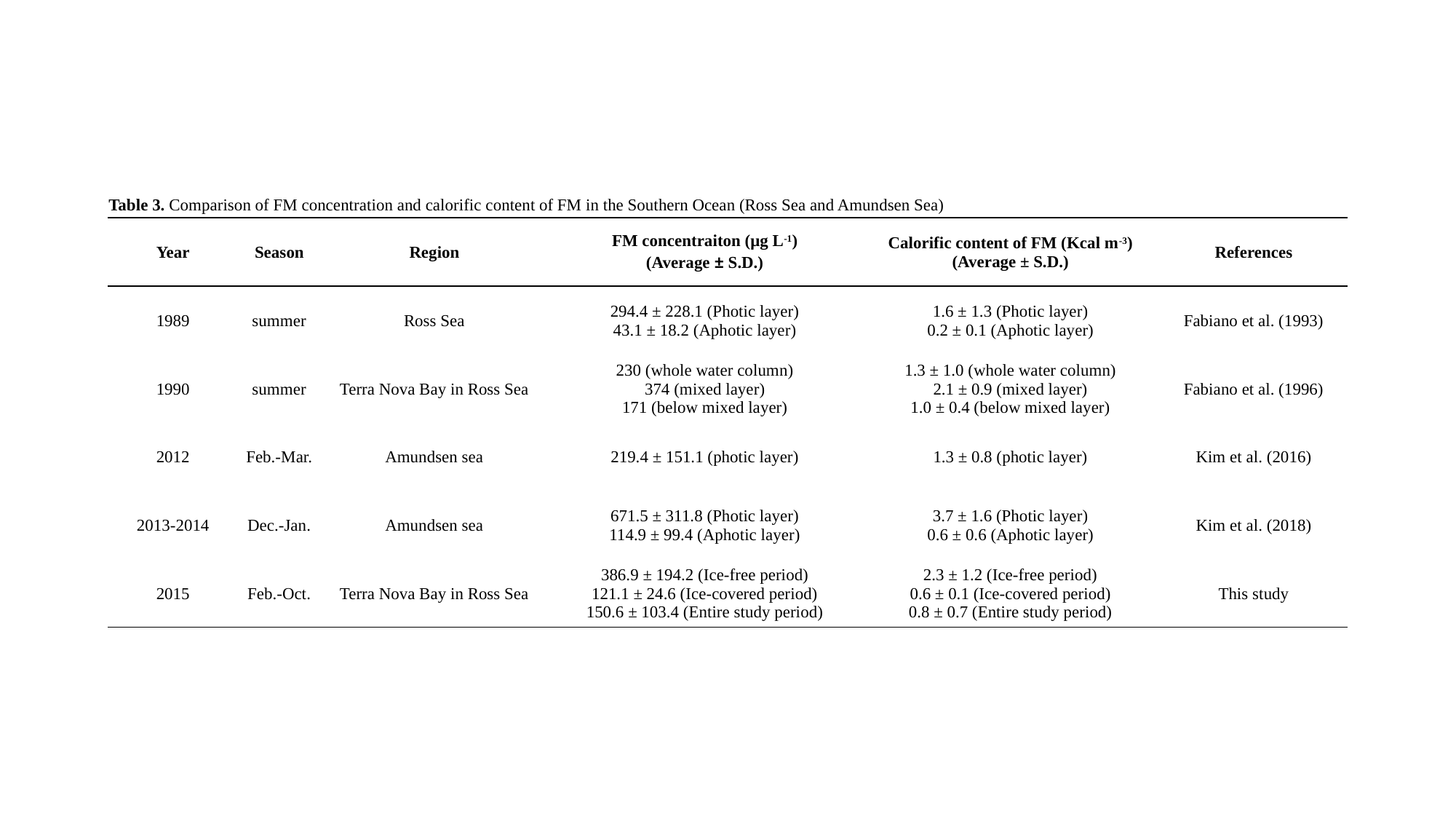

| Table 3. Comparison of FM concentration and calorific content of FM in the Southern Ocean (Ross Sea and Amundsen Sea) | | | | | |
| --- | --- | --- | --- | --- | --- |
| Year | Season | Region | FM concentraiton (μg L-1) (Average ± S.D.) | Calorific content of FM (Kcal m-3) (Average ± S.D.) | References |
| 1989 | summer | Ross Sea | 294.4 ± 228.1 (Photic layer) 43.1 ± 18.2 (Aphotic layer) | 1.6 ± 1.3 (Photic layer) 0.2 ± 0.1 (Aphotic layer) | Fabiano et al. (1993) |
| 1990 | summer | Terra Nova Bay in Ross Sea | 230 (whole water column) 374 (mixed layer) 171 (below mixed layer) | 1.3 ± 1.0 (whole water column) 2.1 ± 0.9 (mixed layer) 1.0 ± 0.4 (below mixed layer) | Fabiano et al. (1996) |
| 2012 | Feb.-Mar. | Amundsen sea | 219.4 ± 151.1 (photic layer) | 1.3 ± 0.8 (photic layer) | Kim et al. (2016) |
| 2013-2014 | Dec.-Jan. | Amundsen sea | 671.5 ± 311.8 (Photic layer) 114.9 ± 99.4 (Aphotic layer) | 3.7 ± 1.6 (Photic layer) 0.6 ± 0.6 (Aphotic layer) | Kim et al. (2018) |
| 2015 | Feb.-Oct. | Terra Nova Bay in Ross Sea | 386.9 ± 194.2 (Ice-free period) 121.1 ± 24.6 (Ice-covered period) 150.6 ± 103.4 (Entire study period) | 2.3 ± 1.2 (Ice-free period) 0.6 ± 0.1 (Ice-covered period) 0.8 ± 0.7 (Entire study period) | This study |
